# Better Design, Better Engagement, Better Data: Adolescents' Insights for Improving Health Research

**DOI:** 10.1111/hex.70549

**Published:** 2026-02-14

**Authors:** Mohammad Karimipour, Mina Fazel, Emma Soneson

**Affiliations:** ^1^ Department of Psychiatry University of Oxford Oxford UK

**Keywords:** adolescents, constructivist grounded theory, data accuracy, engagement, health research, participation, qualitative study, retention

## Abstract

**Introduction:**

Adolescent health research can be hampered by a lack of representative participation, limited long‐term engagement, and inaccuracies in self‐reported responses. This study aimed to explore adolescents' perspectives on participating in health research in order to understand how to enhance engagement, representation, and data quality.

**Methods:**

Forty‐six adolescents (aged 16–18 years) at five English schools participated in nine focus groups held from March to July 2024. Constructivist grounded theory was deployed to explore factors influencing adolescents' participation in health research and the processes underpinning interactions between these factors.

**Results:**

Participants raised five main considerations regarding adolescents' participation in health research. They believed that (1) *positive relationships with researchers* can foster an atmosphere of (2) *emotional security* that reduces the potential influence of (3) *others' judgments* on adolescents' relationships and emotional security. They felt that the more (4) *choice and control* adolescents have throughout the research cycle, the more likely they are to feel secure, which encourages them to remain engaged in research and provide accurate self‐reported data. The combination of *positive relationships with researchers* and *choice and control* was thought to contribute to adolescents perceiving health research as an (5) *influential collaboration*, which empowers them to participate and provide accurate information, especially about their personal life experiences.

**Conclusion:**

This study suggests that enhancing adolescent engagement, representation, and data quality in research is possible but requires a substantial investment of time and resources. Such investment would enable researchers to foster positive relationships with adolescent participants by providing them with choice and control (where feasible) over research processes and by better communicating the potential impact of their participation. Given adolescent concerns about judgment and potential consequences of research participation, they are likely to benefit from clear, accurate, and reassuring conversations on these topics.

**Patient or Public Contribution:**

Two online meetings with a Young People's Advisory Group (YPAG) of adolescents aged 16 to 18 were conducted. In the first, eight participants provided feedback on a mock focus group's structure and content. In the second, they reviewed a conceptual model grounded in the focus group data, highlighting missing elements and aspects that lacked clarity.

## 1 Introduction

Despite positive trends on some indicators of adolescent health, others, such as mental illness, are deteriorating [1, 2, 3, 4, 5, 6]. Both Lancet Commissions on Adolescent Health [7, 8] have advocated for essential investment into adolescent mental health and highlighted the importance of enhancing adolescent participation in health research. However, despite this global recognition and momentum, significant challenges remain in translating these commitments into effective, inclusive, and sustainable adolescent health research.

Obstacles, including lack of trust, barriers to informed and ongoing consent and assent, and limited autonomy throughout the research process, can impact adolescents' decisions of whether and how to be involved in research [9, 10, 11, 12, 13, 14, 15]. Concerningly, recent evidence suggests that some adolescents are more likely than others to face barriers to participation [13], which suggests that concerns about research can reduce both sample size and representativeness [16] as well as increase the burden on those underrepresented adolescents who do choose to participate [12].

Taken together, this evidence suggests that systematic exploration of the barriers and facilitators of meaningful participation in adolescent health research might have dual benefits of improving participant experience as well as the quality of the research itself. In particular, considering adolescents' own perspectives, which is in line with their right to participate in decisions affecting them [17], can support the development and implementation of strategies that promote greater and more meaningful participation. This has the potential to lead to more reliable data, which is essential to inform evidence‐based decision‐making in health, education, and policy [10, 12, 14, 18, 19, 20].

Previous studies exploring adolescents' views on participation in health research have offered insights that, when paired with meaningful study‐specific involvement such as partnerships with Young People's Advisory Groups (YPAGs) or young co‐researchers, can help support more impactful research. For example, a recent study of over 17,000 secondary school students indicated that nearly half of participants who completed a school‐based survey of health and well‐being reported concerns about privacy, confidentiality, and data use [13]. A scoping review of qualitative and mixed‐methods studies [21] reinforced the importance of privacy and confidentiality as potential barriers to research participation, and also highlighted barriers related to negative emotional experiences and the inconvenience of participation. On the other hand, the review indicated that adolescents believe health research participation benefits them by enabling positive emotional experiences, facilitating skill development, promoting relationships (particularly with peers and families), and enhancing their sense of self‐efficacy. These findings highlight important areas that might require additional attention when designing and delivering adolescent health research.

### 1.1 The Current Study

Whilst a growing body of literature has suggested that strategies designed around barriers and facilitators can improve participation and retention in adolescent health research, there is much more to be learned about how to encourage adolescents to meaningfully engage with research [11]. An integrative framework can provide further insight and nuance into how adolescents' concerns, motivations, and emotions interact with one another and the environment to influence decisions about and experiences of participating in health research. The current study therefore aimed to explore adolescents' perspectives on participating in health research in order to understand how to enhance engagement, representation, and data quality. Through developing a grounded theory based on adolescents' perspectives, the study examined how research procedures and systemic, social, and psychological factors can impact meaningful and accurate participation in health research.

## 2 Materials and Methods

### 2.1 Data Source: *The BrainWaves Adolescent Consent Study*

The present study analysed the data collected as part of the *Brainwaves Adolescent Consent Study*, a five‐arm randomised controlled trial (RCT) that examined whether adolescents' participation in health research is influenced by the procedures used to obtain informed consent. Full details of the trial design can be found in the pre‐registration (https://osf.io/8dkpv), but in brief, this mixed methods trial comprised two complementary strands of research: (1) an online survey about health and well‐being in which secondary school students were randomly assigned to one of five procedures for obtaining informed consent and (2) a series of focus groups that explored students' views on consent and the five specific consent procedures studied as well as their broader perspectives on participation and retention in health research.

The study reported in this article presents an additional analysis of data collected from the RCT focus groups. As the trial progressed, it became clear that the richness of the qualitative data around wider questions of participation and retention merited a deeper exploration that extended beyond the more specific questions surrounding research consent (which are presented in the main trial paper).

### 2.2 Methodology

This study deployed Constructivist Grounded Theory (CGT) methodology, which is a primarily inductive methodology based on pragmatism, symbolic interactionism, and constructivism. It enables researchers' reflexive use of their prior knowledge and preconceptions to make sense of participants' various perspectives and construct a theory grounded in data with qualitative explanatory power. Unlike deductive qualitative approaches, CGT allows researchers to construct new concepts based on their interpretations of the data [22, 23]. CGT was adopted to enable deep exploration of adolescents' perspectives, leading to the development of an integrative framework.

### 2.3 Adolescent Involvement

Two online meetings were conducted with a YPAG consisting of a geographically and sociodemographically diverse group adolescents aged 16–18 who advised on various aspects of the wider *BrainWaves Study*. In the first 2‐h meeting, eight adolescents took part in a mock focus group and provided feedback on its structure and content. In the second 1‐h meeting, eight adolescents reviewed the preliminary versions of the conceptual models developed and provided feedback including which aspects made sense, which did not, and whether they believed anything was missing. Members of the YPAG received £15 per hour for their contributions.

### 2.4 Procedures

#### 2.4.1 Recruitment

The *BrainWaves Adolescent Consent Study* aimed to recruit a diverse sample of 16–18 year old students enrolled in English secondary schools and Further Education Colleges (FECs). School recruitment for the overall trial occurred between June 2023 and May 2024 through three primary pathways: (1) known networks of the *BrainWaves Study* team; (2) online outreach, including mailing list advertisements, newsletters, and social media posts; and (3) direct email contact with schools and FECs as part of a targeted outreach campaign. All students aged 16–18 years at enrolled schools were eligible to participate in the trial.

Individual participant recruitment for the focus groups occurred between March and June 2024. Students at all 11 schools and FECs participating in the RCT were eligible to participate in the focus groups, though not all schools and FECs chose to send details to their students (largely due to competing school priorities such as exams). For interested schools, recruitment was facilitated through school staff, who shared an invitation letter, information sheet, and expression‐of‐interest form with eligible students. Although the original aim was to purposively sample students to capture a diverse range of views and experiences, in practice, all students who completed the expression of interest form were contacted with an invitation to participate. Some, but not all, students had participated in the quantitative element of the *BrainWaves Adolescent Consent Study* (i.e. the online survey completed under one of five consent procedures).

### 2.5 Participants

Nine focus groups of three to eight students, six in‐person and three online, were conducted with 46 students from three secondary schools and two sixth‐form colleges (UK institutions preparing adolescents aged 16–19 for higher education) in the English counties of Hertfordshire, Berkshire, Surrey, Kent, and West Yorkshire. Two schools were independent (three groups, 17 students) and three were state‐maintained (six groups, 29 students); four schools were mixed‐gender (seven groups, 34 students) and one was all‐girls (two groups, 12 students). Thirty‐seven participants were in Year 12 (ages 16–17) and nine were in Year 13 (ages 17–18). Fourteen students described themselves as boys, 27 as girls, one as non‐binary, one as a trans boy, and three as uncertain about their gender. Forty students identified as White, three as Asian, one as mixed ethnicity, one as Black, and one as ‘Other ethnicity’.

### 2.6 Focus Groups

The focus groups were held between March and July 2024. The groups were co‐facilitated by MK and ES, who identify as a man (MK) and woman (ES) and who were in their late 20s/early 30s at the time of the focus groups. Both hold doctoral degrees, and in terms of experience, one facilitator (MK) has specific expertise in CGT and the other (ES) has a strong track record of working with young people as well as a comprehensive overview of the topic area. The groups were conducted in‐person on school premises or online via Microsoft Teams, depending on school preferences and availability. Each focus group lasted approximately 90 min, including a 10 min break, and was audio‐recorded. Participants were remunerated for their time with £25 Love2Shop vouchers.

After obtaining written informed consent, the facilitators described the project and agreed ground rules. A fictional overarching narrative of students deciding whether to participate in an annual self‐reported survey was used to illustrate a series of questions about participating in health research, consenting procedures, confidentiality, privacy, data accuracy, and long‐term retention (Supporting A1). As part of this narrative, three hypothetical characters were developed (Supporting A2) based on the characteristics and experiences identified in a complementary study of adolescents' concerns about research participation [13]. The hypothetical characters were accompanied by figurative images generated using GenCraft software [24]. The YPAG had, when consulted, suggested how using characters can facilitate self‐distancing and concrete thinking and therefore empower adolescents from diverse backgrounds to contribute their views. For example, participants' self‐identification with a character might motivate them to share their worries and thoughts. Additionally, the characters were used to prompt consideration of a wider range of issues that might influence meaningful research participation.

To comply with the iterative nature of CGT [22, 23], the focus group process was constantly reviewed and modified to address emerging gaps in the data.

### 2.7 Data Analysis

Audio‐recordings were transcribed using the automatic transcribing feature of Microsoft Word online on the University of Oxford's Office365 platform and subsequently manually checked. Data analysis was carried out by MK using NVivo14 [25]. Line‐by‐line coding of transcripts was done iteratively while conducting further focus groups to identify gaps in the data. Using constant comparative methods, codes and emerging analytical ideas were compared with one another and the data, which enabled in‐depth analysis during which new concepts and categories were developed.

### 2.8 Methodological Self‐Consciousness

In alignment with Charmaz's [22, 23] conceptualisation, this study supported researcher reflexivity throughout the research. Several recommended exercises were utilised, including drafting a positionality statement, engaging in memo‐writing, comparing the final model with the original data, presenting the model to a YPAG, and holding critical and reflexive discussions within the research team.

### 2.9 Ethical Approval

The *BrainWaves Adolescent Consent Study* was approved by University of Oxford Interdivisional Research Ethics Committee (reference number: R86083/RE004).

## 3 Results

### 3.1 Overview

**Figure 1:**
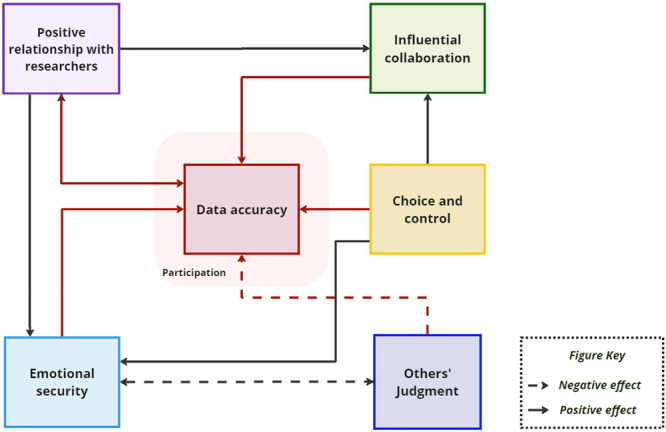
Visual model summarising adolescents’ perspectives on research participation. This model illustrates how the key categories of (1) positive relationships with researchers, (2) emotional security, (3) others’ judgment, (4) choice and control, and (5) influential collaboration all contribute to research participation and data accuracy.

**Figure 2:**
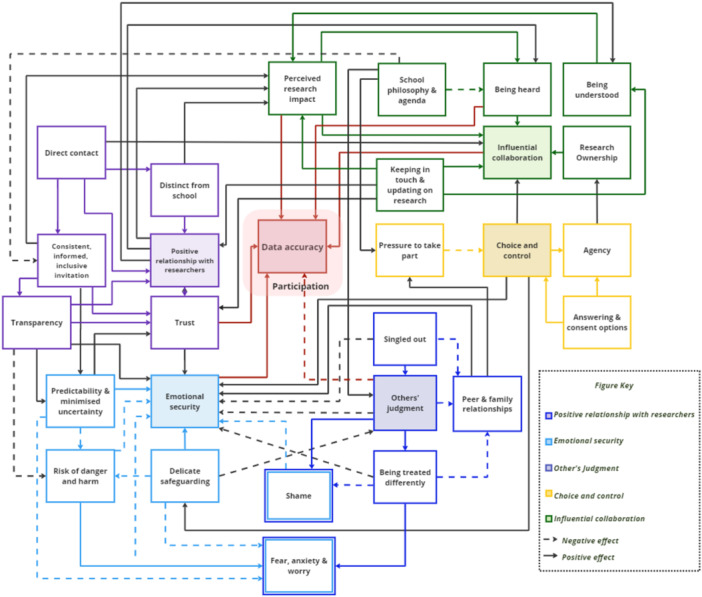
The detailed processes that underpin the main categories, including researchers’ positive relationship with participants, emotional security, the influence of others’ judgment on adolescents’ relationships, choice and control, and influential collaboration.

The visual models were developed to depict factors identified by participants as influencing the quality of adolescents' participation in health and wellbeing research (Figures 1 and 2). Participants suggested that through contacting adolescents directly, providing detailed information, and differentiating the research team from the school, a *positive relationship with researchers* forms, characterised by trust. This relationship contributes to adolescents' *emotional security* within the context of providing self‐reported data, which is also promoted by transparent communication of research procedures, data governance, and safeguarding considerations. This emotional security helps adolescents feel more protected against others' judgment and its influence on their relationships by reducing the potential for relationally aversive emotions and negative feelings of shame, being singled out, loneliness, anxiety, and/or fear. *Choice and control* throughout the research cycle, ranging from consent procedures to safeguarding protocols and actions, support adolescents to participate in research, provide accurate answers, and feel a sense of agency. Choice and control, alongside the perceived bond with researchers, support adolescents to view their contributions as an *influential collaboration* rather than a mandatory task imposed on them (e.g. by schools or researchers). Adolescents' perceived agency can help develop a sense of collective ownership of the research, which in turn validates their perspective. Moreover, based on relationships with researchers and clear explanations of the research and its potential impact, they hope the collaboration will be impactful, especially for other adolescents, motivating them to participate and provide valid responses to the best of their ability. Some examples of participants' ideas for potential ways to improve the experience of taking part in health research are presented in Supporting Material B.

### 3.2 Category 1. Positive Relationship with Researchers

Most participants suggested that direct contact is the first step in adolescents building a *positive relationship with researchers*. Occasionally, participants implied that introduction through schools might be reassuring for some adolescents. However, following initial introductions, participants preferred researchers to independently communicate with them, without continued school involvement. The more researchers can separate their image from that of the school, the more likely adolescents are to value and be interested in the research, leading to more accurate responses. This was viewed as particularly important for sensitive topics that might lead schools to contact parents or take safeguarding/disciplinary action. For this reason, participants were also insistent that individual responses should not be accessible to or monitored by the school.

Participants perceived researchers appearing as ‘normal’, conversing in a friendly way, introducing themselves and their professional background, showing authentic interest in and excitement about the research and its anticipated impact, and encouraging questions as all contributing to relatability and the establishment of a mutually‐beneficial relationship.

I think what is more important is building that personal bond because as [it was] kind of just said, I think having that trust, you are not only more likely to get answers, but also the true answers as well. I think especially, like, [in] an online questionnaire, it's so easy to put on, like, a false personality or whatever, which obviously on [researchers'] behalf, that's not going to give you, like, the best representation.

Participant 3, focus group 6

If you have that relationship and that friendly sort of communication going on, you're more likely to have honest answers.

Participant 1, focus group 8

Participants also valued inclusivity, believing that when researchers ‘go out of their way’ to connect with adolescents from diverse backgrounds and with varied characteristics and experiences, a wider range of adolescents will participate.

Some dyslexic people have to have colour sheets to read, so if you have an option to say what colour you want, it would be easier to read and more accessible to wider audiences… it's like you're going out of your way to be more diverse and get people out there.

Participant 6, focus group 1

### 3.3 Category 2. Emotional Security

**Figure 3:**
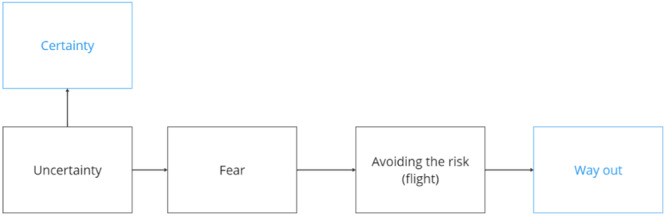
Adolescents’ fears arising from uncertainty about research processes may be alleviated by providing detailed information to address their concerns, emphasising their right to opt out, and/or allowing them to skip certain steps or questions.

Participants noted that positive relationships with researchers, built on trust and transparency, support *emotional security* within research. Uncertainty and unpredictability surrounding procedures and outcomes can cause stress, fear, and anxiety, which may lead adolescents to decline invitations to participate, withdraw from the study, or seek further information for reassurance (Figure 3). Transparent research invitations and positive connections and conversations with researchers can, according to participants, support this emotional security.

I feel like if you have control of this study, you feel more secure in, like, how it's going to be used, and, like, how, like, your answers are gonna affect the study. So, like, if you're in control of it then, like, they're gonna more likely answer all the questions because they know that if they don't feel comfortable with their answers after they've completed the questionnaire, they can withdraw it.

Participant 6, focus group 9

Participants suggested that some adolescents worry about being exposed to triggering and upsetting questions. They emphasised the need for clear communication about safeguarding procedures, noting that poor safeguarding (e.g. lack of clarity upfront or suboptimal execution of required action(s)) can lead to emotional, relational, or even physical harm. They also expressed concern about the legal and disciplinary risks of disclosing sensitive information in research.

Participants noted that the research setting/environment can significantly impact emotional security. Most participants preferred to participate in private settings where their answers could not be inadvertently or deliberately observed by students or staff. Group settings had potential to lead to judgment from peers and staff, making some adolescents feel worried or ashamed about providing accurate answers, especially when they do not feel emotionally secure.

Jayden [the fictitional character with a difficult home life] was doing [a survey] with his friends. And all of his friends, they didn't have much of home difficulties. Nothing at all. And all of them, like, had a perfect home life. And then he was sat there thinking, you know, 'My life isn't like that, but I don't want people to know because I feel ashamed.' He's not gonna give the right answer because of that shame. And the one thing [he wants is] to feel like everyone else.

Participant 1, focus group 6

### 3.4 Category 3. Others' Judgment

A major concern expressed by participants was *others' judgment*, which could affect adolescents' relationships and threaten their emotional security within research. It was noted that some adolescents might worry that if their decisions about participation differed from their peers', they might be singled out and judged. Participants thought that in survey‐based studies, allowing adolescents to choose their level of anonymity could promote autonomy. However, participants raised concerns about the potential for judgment regarding their choice of anonymity.

I think being able to remain completely anonymous will get you the more, like, in‐depth detailed answers so, like, if it was Jayden, he would maybe open up more if he knew, like, none of his [identifying] data was being included in that. And also, obviously not doing it, like, in form around people, that might also affect if you choose to remain anonymous or not, because if a bunch of people on your table [are] like, 'Oh no, why do you need to remain anonymous? That's silly, like, we're not being abused at home', then that might make him feel like, 'Oh, yeah, I don't want to be the odd one out choosing to be anonymous'.

Participant 5, focus group 1

Another concern pertained to the risk of sensitive data being shared with the school, peers, or family, particularly if safeguarding issues were not managed carefully. Participants explained that such breaches could lead to feeling judged, strained relationships, and changes in how they were treated, which in turn might cause feelings of shame, worry, or fear. For instance, participants mentioned how adolescents might not want others to know about self‐harm or bullying or might worry that their data could be monitored by the school, further exacerbating worries about judgement. Additionally, some participants had concerns that peers would also treat them differently, resulting in feelings of shame, worry, or fear. Concerns about potential judgement led some to suggest that adolescents might be more comfortable participating in research with either trusted friends or students unknown to them, rather than with general classmates.

### 3.5 Category 4. Choice and Control

Participants suggested that greater *choice and control* can make adolescents feel more secure, safe, comfortable, and confident to provide accurate answers, whereas direct or indirect pressure to participate from school, peers, or family might have the opposite effect. Participants implied that schools framing research as a mandatory task can also deter genuine participation and data accuracy.

Participants believed that greater choice and control over research procedures and processes (e.g. in terms of questions and response format or level of anonymity) could promote a sense of agency that helps adolescents not perceive research as a one‐sided power dynamic, thereby supporting greater participation and response accuracy. Participants suggested that when adolescents feel pressure to participate, they might provide less accurate answers to ‘regain control’ or put less effort into the process by providing answers they think researchers want to hear, leading to social desirability bias.

The person, like, being questioned during the survey always has, like, full control over their answers […] So, removing their, like, control over how their data is processed might change how they're… how truthful they are about their own answers. Because at the end of the day, they'd still want the control. It's just they'd get it by lying instead of by controlling how the data is published.

Participant 2, focus group 9

### 3.6 Category 5. Influential Collaboration

The relationship between adolescents and researchers, both before and during the research project, was considered to influence adolescents' expectation of being heard and understood as well as their belief that the research could lead to real‐world change. The separation of the research team from the school can reassure some adolescents, as students' perceptions of their school's philosophy and agenda could influence their participation, especially when students believe their school overly values its image and reputation. Participants worried that such schools might be too forceful in encouraging participation and even modify research invitations to fit within school and/or parent ideals. Participants believed that researchers' direct contact and ongoing updates on progress and outcomes–especially in longer‐term studies–can create an atmosphere where adolescents are likely to perceive their contributions as valued and taken seriously.

Participants implied that another factor that strengthens this collaboration is learning about the research's purpose and impact. They suggested that adolescents evaluate participation against their values, and having shared values with the research project and/or team would encourage participation and response accuracy, particularly where adolescents are driven by prosocial motivations. Furthermore, participants suggested that perceived agency helps frame participation as a collaboration rather than a mandatory task and promotes a sense of shared ownership, further motivating meaningful participation. Clear and transparent communication of the goals, processes, progress, strengths, limitations, and potential impact of research helps adolescents understand researchers' perspectives, which encourages them to collaborate more empathetically, especially when a strong researcher‐adolescent bond has been formed.

When someone puts effort into something, they don't want the effort to go to waste […] I guess if it's long, people will feel like, 'Oh it's… I don't want to do that cause it's too long,' but also they might think [the survey] might actually do something because it's time consuming.

Participant 4, focus group 7

In summary, this category–which describes the research process–comprises three main properties: (1) that adolescents' participation is impactful, leading to real‐life improvements for some individuals, particularly those experiencing health or wider difficulties; (2) that researchers acknowledge adolescents' collaborative role by actively listening to and accurately interpreting their perspectives; and (3) that the research process is mutual and collaborative, rather than a one‐sided process in which young people merely provide data.

You could do like updates on how the study is going. […] Something to show, like, the real meaningful impact of the study, not just in 10 years' time but like now, yeah. And then you feel more connected, and you want to keep on going with it because […] it makes them feel like it was worth something. Like, they weren't just another like respondent [but] like an actual person whose, like, words are being valued by the researchers. You know, because then they wanted to do it again and help more.

Participant 1, focus group 6

## 4 Discussion

This qualitative study of 46 adolescents highlights the importance of deliberate and inclusive study design that empowers adolescents to meaningfully engage with research about their health and well‐being. Findings suggest that efforts to foster *positive relationships*, enhance *emotional security*, reduce the *perceived risk of judgment*, offer *choice and control* throughout the research process, and create opportunities for *influential collaboration* can synergistically interact to empower adolescents to participate, remain engaged, and provide more accurate data.

Despite growing public interest and investment in adolescent health research [1, 4, 6, 26], barriers to conducting, and implementing findings from, high‐quality research persist at many levels [27, 28]. Considering the potential impact of these barriers on evidence‐based decision‐making, it is essential to understand the factors that influence (long‐term) participation and data integrity, while also reducing wasted time and effort. Encouragingly, many of the challenges identified in this study can be addressed, albeit with investment of time and resources into what should be considered foundational components of research, such as building and maintaining meaningful relationships with adolescents and communicating clearly and openly with them.

The emphasis on researchers cultivating *positive relationships with young people*, as well as considering the potential influence of *others' judgment* on adolescents' relationships, is consistent with previous findings on the interpersonal elements of conducting research with young people. Others have demonstrated that adolescents hope to benefit from improved interpersonal relationships when participating in research, such as making new friends and interacting with peers [21], highlighting one way to promote engagement that many researchers might overlook. The present study further underscores the importance of adolescents having an opportunity to foster genuine connections with the research team [11, 29], which may benefit the quality of the research, as participants suggested that adolescents' efforts to meet perceived expectations might compromise the validity of their responses or contribute to social desirability bias [30]. Although some studies might be so large that any direct engagement can be difficult, there remains a wide range of ways to engage, for example, through social media or larger engagement events. Moreover, positive relationships between researchers and adolescents can help mitigate the mistrust in health research reported in the literature [11, 13, 29, 31]. In discussing findings with the YPAG, members had many concrete suggestions for building relationships, including researchers communicating their genuine interest in the research, involving adolescents as research partners (e.g. as youth advisors or in the context of a YPAG), and creating a safe and open spaces for adolescents to ask questions or raise concerns.

Consistent with previous evidence regarding adolescents' concerns about research on sensitive topics [21], this study suggests that emotional security in research can be threatened by various factors, particularly others' judgment, which can lead to negative emotions such as shame, anxiety, and fear. Participants highlighted several relational and emotional concerns, including the potential negative consequences of sharing information without consent, inadequate safeguarding procedures, and possible adverse effects on relationships. Some adolescents worried about being singled out or treated differently as a result of their participation or responses, which is not unexpected given that adolescence is a time characterised by a heightened focus on developing complex socialisation skills and emotion regulation [32].

The concept of *influential collaboration* resonates with the findings of an autoethnographic study which found that effective adolescent research involvement (i.e. in an advisory capacity) can be facilitated through (1) commitment motivated by prosociality, individual interests, and a common purpose; (2) elevated inclusiveness and decreased social uncertainty; (3) minimised power imbalances (which also resonates with *choice and control* category); and (4) enhanced representation [33]. Our work extends these findings by suggesting that such facilitators are relevant not only to promoting involvement but also for encouraging meaningful participation. Other findings confirm adolescents' prosocial motivations for taking part in health research [21]. For instance, adolescents have suggested that altruism, reinforced by clear communication of the potential impact of taking part, could convince some adolescents to participate in research, even in the face of other barriers [34, 35]. This study further suggests that, for research that takes part in the school environment, it is important to consider adolescents' perceptions of *why* their school has chosen to take part in research, as these can influence whether adolescents view their contributions as an influential collaboration.

Aligning with pragmatic lessons from prominent adolescent studies [35], the present study suggests that increased choice and control over study processes can promote a greater sense of agency, which helps adolescents manage potential (di)stress and avoid judgment from others. The relative importance of choice and control in research might be heightened during adolescence–a period where individuals transition to being more independent and autonomous. This finding also aligns with well‐established experimental findings and theoretical models suggesting that tasks requiring motivation–such as those involving performance under evaluation–tend to trigger heightened stress responses, especially when perceived as uncontrollable or subject to negative judgment from others [36]. Participants suggested that providing adolescents with choice and control fosters a sense of collaboration, which encourages more active and accurate engagement.

### 4.1 Strengths and Limitations

This study explored a wide range of ideas from students from diverse socio‐demographic backgrounds and with a variety of life experiences. Both facilitators perceived most participants as thoughtfully engaged, though in some groups there were individuals who were less likely to contribute. Nevertheless, the findings might have been influenced by social desirability bias or by the views of those adolescents who were more confident in expressing their opinions and/or were especially interested in research. When interpreting the findings, it is further important to note that students were recruited from schools that actively chose to participate in a study about mental health and well‐being, which may have provided additional context or experiences that influenced students' perspectives. Furthermore, it must be acknowledged that this study explored the perspectives *of a group of adolescents who had consented to take part in a research study* – it is plausible that these adolescents' perspectives and concerns might vary from those of their peers who chose not to participate.

### 4.2 Implications and Future Research Directions

This research provides a framework for understanding adolescents' perspectives on meaningful participation in health research. Alongside other empirical evidence and study‐specific adolescent involvement, the findings can guide researchers in how to improve (long‐term) participation and data accuracy. The findings suggest a need for further investigation into the emotional and relational dimensions of adolescents' experiences of health research, particularly from the perspectives of marginalised and minoritised groups, who may face additional or amplified barriers to participation and long‐term engagement [11]. Further research could also explore the perspectives of school staff, researchers, and parents, which may provide complementary insights and help expand the concepts discussed.

## 5 Conclusions

The findings of this study suggest that giving balanced attention to both *research processes* and *adolescent emotional and relational dynamics* may foster sustained, meaningful, and accurate engagement within the context of adolescent health research.

## Author Contributions

**Mohammad Karimipour:** conceptualisation, methodology, investigation, data curation, formal analysis, visualisation, writing – original draft. **Mina Fazel:** funding acquisition, conceptualisation, supervision, writing – review and editing. **Emma Soneson:** funding acquisition, conceptualisation, methodology, investigation, project administration, supervision, writing – review and editing.

## Ethics Statement

The *BrainWaves Adolescent Consent Study* was approved by the University of Oxford's Medical Science Interdivisional Research Ethics Committee (reference number: R86083/RE004). Written informed consent was obtained from all participants prior to the focus groups, and potential participants were given sufficient time to decide whether they wanted to take part in the study.

## Conflicts of Interest

The authors declare no conflicts of interest.

## Declaration of Generative AI and AI‐Assisted Technologies in the Writing Process

During the preparation of this work, OpenAI's ChatGPT was used to improve the readability and language of the manuscript. After using this tool, all authors carefully reviewed and edited the content as needed and take full responsibility for the content of the published article.

## Supporting information

Suplementary Materials R1.

## Data Availability

In accordance with our ethical approvals, applications for access to de‐identified focus group transcripts may be submitted via the BrainWaves Data Hub. All applications are subject to approval from the BrainWaves Data Access Committee. For more details, visit https://brainwaveshub.org/for-research/.
