# Supplementary material for: Better Design, Better Engagement, Better Data: Adolescents' Insights for Improving Health Research
Source: Health Expect. 2026 Feb 14;29(1):e70549. doi: 10.1111/hex.70549 (PMC12906294; doi:10.1111/hex.70549)
Supplement: Supplementary file 1 — Suplementary Materials R1. [file HEX-29-e70549-s001.docx]

**Supplementary Materials**

**A) Focus Group Materials**

**A.1 Initial Questions *(Contextualised within a fictional narrative of students deciding whether to take part in a health and well-being survey)***

*N.B. These questions were continuously revised according to the principles of constructivist grounded theory to address gaps identified in the data.*

1. What information should students receive about the research study to help them decide whether to take part?
   1. Is it useful to get information about the research team? Why or why not?
2. What concerns might adolescents have about taking part? (As a reminder, you can answer generally or imagine what concerns your character might have about taking part.)
   1. How might these be addressed?
   2. [If nothing raised about confidentiality/privacy] What concerns, if any, might adolescents have about confidentiality and privacy?
   3. [If nothing raised about data use] What concerns, if any, might adolescents have about how their information is used?

**[Participants shown an animation explaining the five consent procedures used in the RCT]**

1. What’s good about each of these pathways? What’s not so good?
2. Let’s rank these on which consent pathways you think students would prefer, with 1 being the most preferred and 5 the least preferred.
3. What might influence whether adolescents answer the questions accurately? Remember you can answer generally or with your character in mind.
4. Do you think that the way researchers get consent might impact how they answer?
   1. Which of the consenting pathways might help students to answer more accurately?
   2. How does that consenting pathway address their concerns?
5. How might we keep adolescents involved over time, so that they’ll want to continue to do the yearly surveys?
   1. Which consent pathway do you think would motivate students to stay involved in the next rounds of the research? Why?
   2. Which would *discourage* them? Why?

**A.2 Fictional Characters**


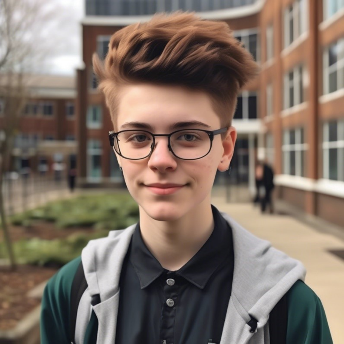

SAM is in Year 12. They identify as gender non-binary. They’ve come out to some of their friends, but not to their teachers or parents.


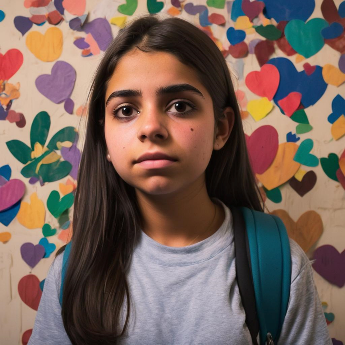


ELLA is in Year 13. She struggles with her mental health and meets often with the counsellor at her school.


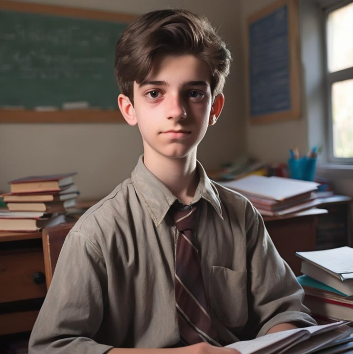


JAYDEN is in Year 10. He has a difficult home life, and his parents fight a lot with each other. Sometimes these fights get violent.

**B) Examples of Participants’ Ideas Regarding the Research Process**

| **Potential considerations for designing adolescent health research** |
| --- |
| 1. Express genuine interest and excitement about the research to motivate adolescents to participate. 2. Meet with adolescents in-person (e.g., group, class, assembly) rather than via virtual meetings; consider recording an introductory video. 3. Inform participants of researchers’ credentials, previous work, and affiliated institutions to build credibility. 4. Highlight the research team’s credibility, as some adolescents trust teams more than individual researchers, believing they are less prone to bias. 5. Involve individuals with a history of mental health challenges to promote relatability and foster a non-judgmental environment. 6. Hold open discussions for concerns and questions, explaining how data will be used and how safeguarding issues will be addressed. 7. Offer a combination of brief introductions to the research (video or bullet points) alongside an expanded information sheet to avoid overwhelming participants. 8. Begin with less sensitive questions to ease adolescents into the research without requiring immediate sharing of more personal experiences. 9. Explain the potential and ongoing real-world impacts of the research to motivate participation and show its broader implications. 10. Clarify how the research differs from other school questionnaires to distinguish its purpose. 11. Consider reducing age gaps between researchers and participants to help adolescents feel comfortable and avoid the pressure to meet expectations. |
